# Supplementary material for: Sustained-input switches for transcription factors and microRNAs are central building blocks of eukaryotic gene circuits
Source: Genome Biol. 2013 Aug 23;14(8):R85. doi: 10.1186/gb-2013-14-8-r85 (PMC4054853; doi:10.1186/gb-2013-14-8-r85)
Supplement: Additional file 5 — HTML Browsable Motif Output. Zipped folder containing all WaRSwap and FANMOD motif output, viewable in a web browser. [file gb-2013-14-8-r85-S5.ZIP › HTML_browsable_motif_output/WaRSwap_all_tair9/sigs_WSWRSLDraw-2000_all.pvals.heatmaps.html/motif_id_238_011101111_tftype_all_upstream_-3000_0.html]

```
BG_MODEL = Rnew2
MOTIF_ID = 238_011101111
TF_TYPE = all
UPSTREAM = -3000_0


PVals
FN_0.2	FN_0.4	FN_0.6	FN_0.8
dg_60.genes	0.167	0.002	0.062	0.001
dg_70.genes	0.084	0.001	0.046	0.001
dg_80.genes	0.462	0.049	0.018	1

ZScores
FN_0.2	FN_0.4	FN_0.6	FN_0.8
dg_60.genes	0.846	4.944	-0.245	-0.032
dg_70.genes	1.366	6	-0.209	-0.032
dg_80.genes	-0.464	1.133	-0.134	NA

StDevs
FN_0.2	FN_0.4	FN_0.6	FN_0.8
dg_60.genes	4.323	1.41	0.29	0.032
dg_70.genes	3.516	1.191	0.244	0.032
dg_80.genes	1.637	0.623	0.138	NA
```
